# Transglutaminase 2 Overexpression in Tumor Stroma Identifies Invasive Ductal Carcinomas of Breast at High Risk of Recurrence

**DOI:** 10.1371/journal.pone.0074437

**Published:** 2013-09-13

**Authors:** Jasmeet Assi, Gunjan Srivastava, Ajay Matta, Martin C. Chang, Paul G. Walfish, Ranju Ralhan

**Affiliations:** 1 Alex and Simona Shnaider Laboratory in Molecular Oncology, Samuel Lunenfeld Research Institute, Mount Sinai Hospital, Joseph and Wolf Lebovic Health Complex, Toronto, Ontario, Canada; 2 Department of Pathology and Laboratory Medicine, Mount Sinai Hospital, Toronto, Ontario, Canada; 3 Department of Laboratory Medicine and Pathobiology, University of Toronto, Toronto, Ontario, Canada; 4 Joseph and Mildred Sonshine Family Centre for Head and Neck Diseases, Mount Sinai Hospital, Toronto, Ontario, Canada; 5 Department of Medicine, Endocrine Division, Mount Sinai Hospital and University of Toronto, Toronto, Ontario, Canada; 6 Department of Otolaryngology – Head and Neck Surgery, Mount Sinai Hospital, Toronto, Ontario, Canada; University of Texas MD Anderson Cancer Center, United States of America

## Abstract

**Introduction:**

Molecular markers for predicting breast cancer patients at high risk of recurrence are urgently needed for more effective disease management. The impact of alterations in extracellular matrix components on tumor aggressiveness is under intense investigation. Overexpression of Transglutaminase 2 (TG2), a multifunctional enzyme, in cancer cells impacts epithelial mesenchymal transition, growth, invasion and interactions with tumor microenvironment. The objective of our study is to determine the clinical relevance of stromal TG2 overexpression and explore its potential to identify breast cancers at high risk of recurrence.

**Methods:**

This retrospective study is based on immunohistochemical analysis of TG2 expression in normal breast tissues (n = 40) and breast cancers (n = 253) with clinical, pathological and follow-up data available for up to 12 years. TG2 expression was correlated with clinical and pathological parameters as well as disease free survival (DFS) of breast cancer patients.

**Results:**

Stromal TG2 overexpression was observed in 114/253 (45.0%) breast cancer tissues as compared to breast normal tissues. Among invasive ductal carcinomas (IDC) of the breast, 97/168 (57.7%) showed strong TG2 expression in tumor stroma. Importantly, IDC patients showing stromal TG2 accumulation had significantly reduced DFS (mean DFS = 110 months) in comparison with patients showing low expression (mean DFS = 130 months) in Kaplan-Meier survival analysis (p<0.001). In Cox multivariate regression analysis, stromal TG2 accumulation was an independent risk factor for recurrence (p = 0.006, Hazard’s ratio, H.R. = 3.79). Notably, these breast cancer patients also showed immunostaining of N-epsilon gamma-glutamyl lysine amino residues in tumor stroma demonstrating the transamidating activity of TG2.

**Conclusions:**

Accumulation of TG2 in tumor stroma is an independent risk factor for identifying breast cancer patients at high risk of recurrence. TG2 overexpression in tumor stroma may serve as a predictor of poor prognosis for IDC of the breast.

## Introduction

Breast cancer is the leading cause of cancer in women with an estimated 1,383,500 new cases and 458,400 deaths worldwide [1], [2]. Despite improvements in treatment strategies recurrence rates are still high among breast cancer patients [1], [2]. This may be attributed to heterogeneous nature of breast cancers representing varied morphologic and biological features, behavior, and response to therapy [3], [4]. Even among breast tumors of similar histologic type and grade, prognosis varies. The clinical decisions for management of breast cancer patients rely on the availability of robust well validated clinical and pathologic prognostic factors to support treatment related decision making [5]. Routine physical examinations along with imaging, histopathological analysis and clinical parameters (tumor size, lymph node status, stage and grade) largely impact the management of breast cancer patients [5]. Currently, breast cancer prognosis assessment methods have limited accuracy, are expensive, and in 20–30% of cases lead to over-treatment with adverse effects. None of the currently known prognostic factors has the ability to predict accurately which breast cancer patients are at high risk of recurrence. Thus, there is an increasing need for identification and validation of prognostic markers for assessment of risk for disease recurrence in breast cancer patients.

Tumors are characterized by alterations in the epithelial and stromal components, which both contribute to disease progression. Recent reports demonstrate synergy between stromal and epithelial interactions, even at the initial stages of breast carcinogenesis, appears necessary for the acquisition of malignancy and provides novel insights into where, when, and how the tumor stroma develops, allowing development of new molecular markers and therapeutic targets [6]. It is now well recognized that stromal cells within and surrounding pathologic lesions also actively contribute to malignant phenotypes through elevated expression of cytokines and growth factors [7]–[12]. They exert their effects through increased deposition and remodelling of the extracellular matrix (ECM). The clinical impact of changes in ECM on tumor aggressiveness and disease outcome needs in depth investigation.

Transglutaminase 2 (TG2), a member of multifunctional enzyme family, modifies glutamine residues by cross-linking proteins, demonstrates protein disulphide isomerase and kinase activities, mediates transmembrane signal transduction and interacts with cell surface and extracellular matrix proteins [13], [14]. TG2 overexpression has been reported in cytoplasm, nucleus, membrane or ECM in tumor cells [13], [14]. Increased expression of cytoplasmic TG2 is associated with increased cell survival, anchorage-independent growth, loss of cell polarity, increased invasion and resistance to chemotherapy in mammary epithelial cells [15], [16]. TG2 promotes tumor progression by initiating a comprehensive program of de-differentiation by inducing epithelial mesenchymal transition (EMT) and cancer stem cell like phenotype [14], [17]–[19]. The resulting tumors remain dependent on TG2-regulated pathways for their growth and survival. Increased TG2 induces expression of transcription repressors including Snail1, Twist, Zeb1, and Zeb2, the key regulators in development of EMT phenotype in cancers [14], [17]–[21]. TG2 overexpression results in constitutive activation of NFKB, the inflammatory transcription factor known to regulate various genes involved in cancer initiation and progression [22], [23]. Nuclear TG2 in association with pRb, p53 and histones regulates cellular functions [24]–[26]. Cell surface TG2 in association with β-integrins serves as a co-receptor for integrin-mediated binding to fibronectin (Fn), thereby regulating cellular adhesion, spreading, motility and survival [13], [14]. Extra-cellular matrix TG2 regulates cell–matrix interactions [27], [28]. TG2 serves as a signalling molecule transmitting signals from outside the cell through Alpha1B adrenergic receptors to a downstream cytoplasmic target, phospholipase C, through hydrolysis of GTP [29], [30]. These findings suggest differential localization of TG2 in cancer cells impacts tumor development, growth, survival or invasion by different cellular mechanisms.

Till date, most investigations on determining clinical relevance of TG2 overexpression in epithelial malignancies including breast cancer are limited to its expression in cytoplasm of tumor cells. However, studies demonstrating an association of TG2 overexpression in ECM with disease recurrence (locoregional/metastasis) are lacking. In this study, we focussed on evaluating the prognostic significance of TG2 overexpression in ECM in breast cancer patients. Further, to evaluate the crosslinking i.e. transamidating activity of TG2 (stroma/cytoplasm), we determined the expression of N-epsilon gamma-glutamyl lysine amino residues (to detect any potential TG2-mediated protein crosslinking events) in the same cohort of the breast cancers using immunohistochemistry. In addition, we stained representative tissue sections (where TG2 is expressed in the stroma) with anti-phospho-FAK or anti-phospho-ERK antibodies to evaluate the effect of stromal TG2 on activation of integrin dependent downstream signaling in breast cancer tissues.

## Materials and Methods

### Patients, Clinicopathological Data Collection and Tumor Specimens

The study was approved by Mount Sinai Hospital Research Ethics Board, Toronto, Canada. Written informed consent was obtained for the acquisition and use of patient tissue samples and anonymized clinical data. The breast cancer database maintained in the Department of Pathology and Laboratory Medicine (PLM), Mount Sinai Hospital (MSH), Toronto, Canada was reviewed for the last 12 years to select breast cancer cases wherein complete clinical, pathological and follow up data were available. Tissue specimens were retrieved from the archived blocks of 253 breast cancer patients (mean age: 59 years; range: 29 to 89 years) undergoing curative cancer surgery during the period 2000–2002. Comprehensive clinicopathologic data were available in digital databases for each of these cases including demography, clinical tumor staging (American Joint Committee on Cancer staging guidelines), surgical; histological grade; recurrence including local, regional, locoregional or distant; treatment, subsequent management and disease status at last clinical review. The hematoxylin and eosin (H & E) stained slides of these cases were reviewed and tumor tissues confirmed by the pathologist (MC). These 253 breast cancer cases were classified as ductal carcinoma in situ (DCIS, n = 60), invasive ductal carcinomas (IDC, n = 168), invasive lobular carcinoma (ILC, n = 16) and invasive mucinous carcinoma (IMC, n = 9). In addition, archived blocks of normal breast tissues (n = 40) obtained from patients undergoing breast reduction surgery were retrieved from MSH tissue bank.

### Treatment and Follow-up

Breast cancer patients (n = 253) were treated with a primary surgery i.e. either breast conserving surgery (BCT), or a mastectomy, as per the hospital protocol. Breast cancer patients who were ER^+^/PR^+^ were given hormonal treatment. Pre-menopausal women were given tamoxifen as their primary treatment option. Post-menopausal patients were given an option of using tamoxifen followed by aromatase inhibitors, which included anastrozole, letrozole, and exmestane. Patients were given tamoxifen for 5 years and then an aromatase inhibitor for 5 years for risk reduction. Patients who received BCT were treated with radiation therapy (RT). Radiation therapy was given from 40 Gy to 50 Gy in fractions of 1.8 to 2.0 Gy. Patients receiving adjuvant chemotherapy (CT) were defined as patients, who were ER^−^/PR^−^ with a tumor size of <0.5 cm, patients who were node negative with a tumor size >2 cm, and patients who had a positive nodal status. These patients were given CT regimens regardless of histology grade, and tumor size. Patients with rapidly progressive disease or visceral crisis received combination chemotherapy (CT) including AC (doxorubicin, cyclophosphamide)/CEF (cyclophosphamide, epirubicin, 5-flourouracil)/CMF (cyclophosphamide, methotrexate, 5-fluorouracil)/FAC (5-fluorouracil, doxorubicin, cyclophosphamide). Patients with metastatic disease were treated with single agents (doxorubicin, docetaxel or paclitaxel).

Follow-up data were available for all 253 breast cancer patients. Survival status, loco-regional relapse or distant metastasis of the breast cancer patients was verified and updated from the records of the Tumor Registry, Mount Sinai Hospital (MSH), Toronto, Canada as of August, 2012. Breast cancer patients were monitored for a maximum period of 143 months (range: 4–143 months; mean 83.9 months and median 93 months). The patients were reassessed on a regular basis and the time to recurrence was recorded. If a patient died, the medical history, clinical examination, and radiological evaluation were used to determine whether the death had resulted from recurrent cancer (relapsing patients) or from an unrelated cause. Disease-free survivors were defined as patients free from clinical and radiological evidence of local, regional, or distant relapse at the time of the last follow-up. Disease-free survival (DFS) was evaluated in the present study for statistical analysis. Disease-free survival was expressed as the number of months from the date of surgery to loco-regional relapse or till date distant metastasis was diagnosed.

### Immunohistochemistry (IHC)

Serial paraffin embedded tissue sections (4 µm thickness) were deparaffinized in xylene, hydrated through graded alcohol series, pre-treated in a microwave oven for 15 min in Tris-EDTA (0.1 M, pH = 9.0) containing Tween 20 (0.05% v/v) for antigen retrieval [31]. Slides were washed with Tris-buffered saline (TBS, 0.1 M, pH = 7.2) containing Triton X-100 (0.1%) followed by treatment with 0.3% H_2_O_2_ at room temperature for 10 minutes to block the endogenous peroxidase activity. Thereafter, sections were incubated with normal horse serum (10%) prepared in 5% bovine serum albumin (BSA) to preclude any non-specific binding. The sections were incubated with either TG2 antibody (mouse mAb cat # MS-300-PABX, 1∶4,000 dilution, Lab Vision Corporation, Fremont, CA)/N-epsilon gamma-glutamyl lysine amino residues antibody (mouse mAb cat # ab424, Abcam, Cambridge) for 60 minutes/anti-FAK (phospho Y397) antibody (rabbit pAb cat # ab4803, Abcam, Cambridge)/anti-ERK1+ERK2 (phospho T202+ T185+ Y187) antibody (rabbit mAb cat # ab32538, Abcam, Cambridge). Slides were washed with Tris-buffered saline (TBS, 0.1 M, pH = 7.2) containing Triton X-100 (0.1%) followed by incubation with biotinylated secondary antibodies for 20 minutes. The sections were finally incubated with VECTASTAIN Elite ABC Reagent (Vector labs, Burlingame, CA) and diaminobenzidine was used as the chromogen. All procedures were carried out at room temperature unless otherwise specified. Slides were washed with Tris-buffered saline (TBS, 0.1 M, pH = 7.4), 3–5 times after every step. Finally, the sections were counterstained with Mayer’s hematoxylin and mounted with D.P.X mountant. In negative control tissue sections, the primary antibody was replaced by isotype-specific non-immune mouse IgG. The sections were evaluated by light microscopic examination.

### Evaluation of Immunohistochemical Staining

IHC scoring was performed under supervision of the pathologist (MC). Immunopositive staining was evaluated in five pathological areas of the tissue sections as described earlier [31]. Immunostaining for all the proteins in this study was evaluated independently in tumor cell cytoplasm, nucleus and stroma by the intensity and percentage of positive staining. Sections were scored as positive if TG2/N-epsilon gamma-glutamyl lysine amino residues/anti-FAK (phospho Y397)/anti-ERK1+ERK2 (phospho T202+ T185+ Y187) immunostaining was observed in the tumor cell cytoplasm or in the stroma when observed by two evaluators (JA & GS) who were blinded to the clinical outcome. These sections were scored as follows: 0, <10% cells; 1, 10–30% cells; 2, 31–50% cells; 3, 51–70% cells; and 4, >71% cells showed immunoreactivity. Sections were also scored semi-quantitatively on the basis of intensity as follows: 0, none; 1, mild; 2, moderate; and 3, intense. Finally, a total score (ranging from 0 to 7) was obtained by adding the scores of percentage positivity and intensity for each of the breast cancer tissue sections. This integrated scoring has proven to work well in our previous investigations [31].

### Statistical Analysis

The IHC data was subjected to statistical analysis using SPSS 20.0 software (SPSS, Chicago, IL) and GraphPad Prism 5.0 software (GraphPad Software, La Jolla, CA). Scatter plots were used to determine the distribution of total score of cytoplasmic or stromal TG2 expression in all tissues examined. The p-value <0.05 was considered significant for statistical analysis [31]. The cut-off of IHC score ≥3.0 for cytoplasmic/stroma TG2 immunostaining was considered as overexpression for further analysis. For N-epsilon gamma-glutamyl lysine amino residues immunostaining, the cut-off of IHC score ≥2.0 for cytoplasmic/stroma was considered as overexpression for further analysis. Expression data thus generated was analyzed to determine significant correlations between TG2 overexpression, clinical parameters and prognosis of breast cancer patients. The correlation of TG2 expression with patient survival (i.e. disease free survival) was evaluated using life tables constructed from survival data with Kaplan-Meier plots as described earlier [31]. Multivariate analysis was carried out using Cox regression models to determine the performance of TG2 overexpression as a marker in comparison to other clinical and pathological prognostic parameters including age, histological grade, tumor size, stage, grade and nodal status of breast cancer patients.

## Results

### Immunohistochemical Analysis of TG2 Expression in Breast Cancer

**Figure 1:**
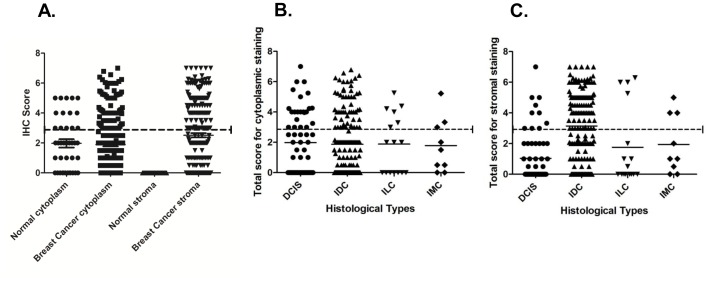
Scatter plot analysis. All breast tissue sections used for TG2 immunostaining were scored on the basis of % positivity and intensity. The total score was calculated as sum total of scores for % positivity and intensity as described in Materials and Methods. Panel A shows the score distribution of TG2 (cytoplasm/stroma) in breast normal and cancer tissues. Panel B and C shows the score distribution of TG2 among different histological types of breast cancer in cytoplasm and stroma respectively.

**Figure 2:**
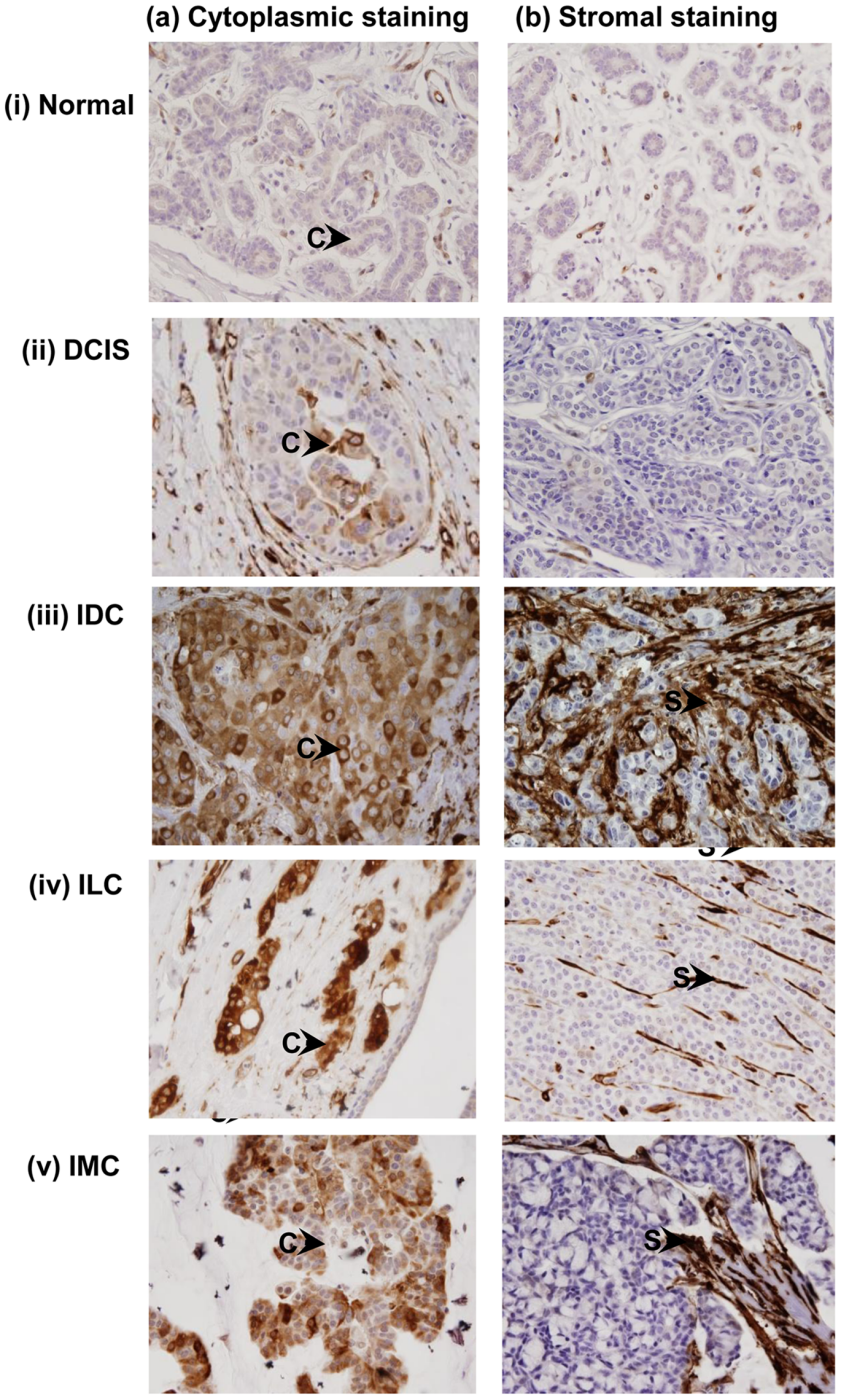
Immunohistochemistry of TG2 in breast cancer tissues. Panel shows (a) cytoplasmic TG2 immunostaining in (i) Normal (ii) DCIS, (iii) IDC, (iv) ILC; and (v) IMC. Panel (b) shows (i) Normal and (ii) DCIS with no TG2 immunostaining in stroma. Strong TG2 immunostaining was observed in tumor stroma of: (iii) IDC, (iv) ILC and (v) IMC. Arrows show cytoplasmic (C) TG2 staining and stromal (S) TG2 staining (Original Magnification X400).

To determine the clinical significance of TG2 overexpression in cytoplasm/stroma, immunohistochemistry was performed in breast normal (n = 40) and cancer tissues (n = 253). Scatter plot analysis shown in Figure 1(A–C) depicts the distribution of IHC scores for TG2 immunostaining in breast normal and cancer tissues. Of the 40 breast normal tissues, 14 cases (35%) showed weak to moderate immunostaining for TG2 in cytoplasm of epithelial cells (Figure 2(i), a, Table 1). However, no TG2 immunostaining was observed in stroma of the breast normal tissues used in this study (Figure 2(i), b, Table 1). Immunohistochemical analysis of 253 breast cancers revealed 199 cases (78.6%) showing strong TG2 immunostaining either in cytoplasm (33.6%) or stroma (45.0%, Table 1). Among DCIS, 22 of 60 (36.7%) showed cytoplasmic TG2, while majority of the cases (50/60; 83.3%) showed no detectable TG2 expression in stroma (Figure 2(ii), a and b, Table 1). Fifty four of 168 (32.1%) IDCs showed cytoplasmic TG2, while 97 cases (57.7%) showed TG2 expression in stroma (Figure 2(iii), a and b, Table 1). Of 16 invasive lobular carcinomas, 6 (37.5%) showed cytoplasmic TG2, while only 4 cases (25.0%) showed TG2 overexpression in stroma (Figure 2(iv), a and b, Table 1). Among IMCs analyzed in this study, 3 of 9 (33.3%) showed cytoplasmic TG2 while only 3 cases (33.3%) showed TG2 expression in stroma (Figure 2(v), a and b, Table 1). Negative control sections, wherein primary antibody was replaced by isotype IgG, no immunostaining was observed in cytoplasm/stroma of breast cancer tissue sections (data not shown).

**Table 1:** Transglutaminase 2 (TG2) expression in cytoplasm and stroma in breast cancer.

Clinicopathological parameters	Total no. of cases	TG2 (Cytoplasm)		TG2 (Stroma)	
	n	n	%	n	%
**Normal Breast**	**40**	**14**	**(35.0)**	**0**	**(0.0)**
**Breast cancer**	**253**	**85**	**(33.6)**	**114**	**(45.0)**
DCIS	60	22	(36.7)	10	(16.7)
IDC	168	54	(32.1)	97	(57.7)
ILC	16	6	(37.5)	4	(25.0)
IMC	9	3	(33.3)	3	(33.3)

* The cut-off of IHC score ≥3.0 for cytoplasmic/stromal TG2 immunostaining was considered as overexpression for further analysis.

**Figure 3:**
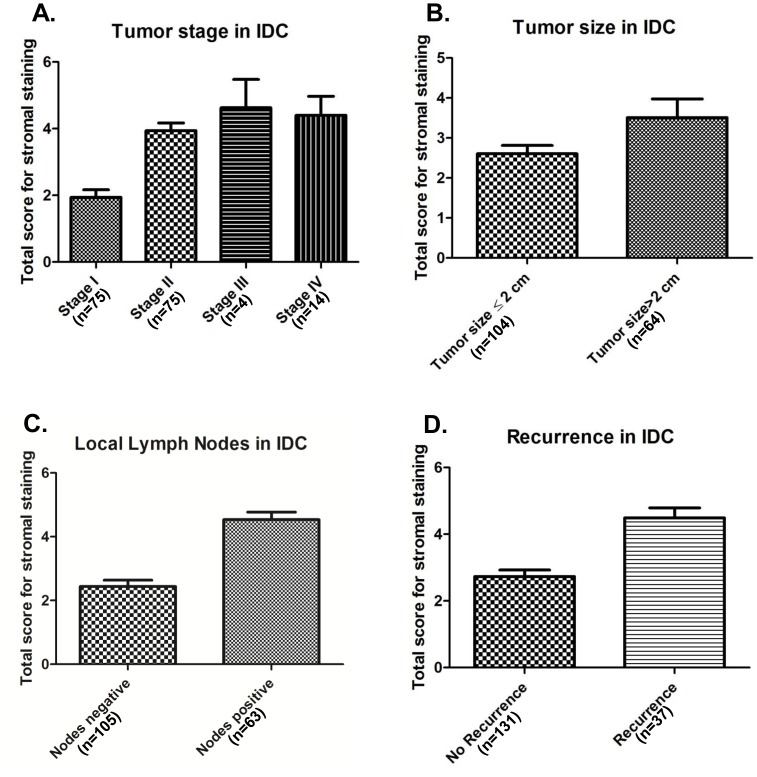
Box plot analysis. Panel (**A**) depicts the IHC scores for stromal TG2 staining in stage I (n = 75, mean score = 1.94), stage II (n = 75, mean score = 3.94), stage III (n = 4, mean score = 4.625) and stage IV (n = 14, mean score = 4.40) IDCs; Panel (**B**) depicts the increase in IHC scores of TG2 immunostaining in stroma with tumor size, tumor size >2 cm (n = 64, mean score = 3.50) as compared to IDC cases with tumor size ≤2 cm (n = 104, mean score = 2.60); Panel (**C**) shows increased TG2 stromal staining in 63 IDCs with positive lymph nodes (mean score = 4.53) as compared to 105 cases with negative lymph nodes (mean score = 2.43); Panel (**D**) shows the increase in IHC scores of TG2 stromal staining in IDCs with recurrence (loco-regional recurrence/distant metastasis ), (n = 37, mean score = 4.49) as compared to IDCs without recurrence, (n = 131, mean score = 2.73).

Box plot analysis revealed significant increase in stromal TG2 with advancing stage (p = 0.020), tumor size (p<0.001), lymph node metastasis (p<0.001) and recurrence (loco-regional recurrence/distant metastasis) (p<0.001) (Figure 3A–3D respectively; Table 2).

**Table 2:** Transglutaminase 2 (TG2) expression in cytoplasm and stroma of breast invasive ductal carcinoma (IDC).

Clinicopathologicalparameters	Total no. of cases	TG2 (Cytoplasm)		p-value	Odd’s ratio (95% C.I.)	TG2 (Stroma)		p-value	Odd’s ratio (95% C.I.)
	n	n	(%)			n	(%)		
**IDC**	168	54	(32.1)	–	–	97	(57.7)	–	–
**Age**									
<59 yrs	**84**	**33**	**(39.3)**			52	(61.9)		
≥59 yrs	**84**	**21**	**(25.0)**	**0.047**	**0.5(0.3–1.0)**	45	(53.6)	0.274	0.7 (0.4–1.3)
**Tumor Size**									
≤2 cm	104	34	(32.0)			**47**	**(44.7)**		
> 2cm	64	20	(31.2)	0.915	1.0 (0.5–1.9)	**50**	**(78.1)**	**< 0.001**	**4.4 (2.2–9.0)**
**T-stage**									
T_1_+T_2_	160	52	(32.5)			90	(56.2)		
T_3_+T_4_	8	2	(25.0)	0.658	0.7 (0.1–3.6)	7	(87.5)	0.081	5.4 (0.7–45.3)
**Nodal Status**									
N_x+0_	105	35	(33.3)			**46**	**(43.8)**		
N_1–3_	63	19	(30.2)	0.670	0.9 (0.4–1.7)	**51**	**(81.0)**	**< 0.001**	**6.5 (2.6–11.4)**
**Stage**									
I+II	150	49	(32.7)			**82**	**(54.7)**		
III+IV	18	5	(27.8)	0.675	0.8 (0.3–2.4)	**15**	**(83.3)**	**0.020**	**4.1 (1.2–15.0)**
**Grade** a									
I	35	9	(25.7)			**13**	**(37.1)**		
II+ III	130	43	(33.6)	0.328	1.5 (0.6–3.5)	**83**	**(63.8)**	**0.004**	**3.0 (1.4–6.5)**
**Distant Metastasis**									
No	144	45	(31.2)			**76**	**(52.8)**		
Yes	24	9	(37.5)	0.544	1.3 (0.5–3.2)	**21**	**(87.5)**	**< 0.001**	**6.3 (1.8–21.9)**
**ER/ PR status** b									
ER^+^	127	39	(30.7)			**68**	**(53.5)**		
ER^−^	32	13	(40.6)	0.285	0.7 (0.3–1.4)	**24**	**(75)**	**0.028**	**0.4 (0.2–0.9)**
ER^+^PR^+^	96	33	(34.4)			**51**	**(53.1)**		
ER^−^PR^−^	32	13	(40.6)	0.523	0.8 (0.3–1.7)	**24**	**(75.0)**	**0.030**	**0.4 (0.2–0.9)**

a Tumor Grades were available for 165 IDCs only;

b ER status was available for 159 IDCs only, in our clinical databases.

### Potential of TG2 as a Marker for Breast Cancer Recurrence and Distant Metastasis

**Figure 4:**
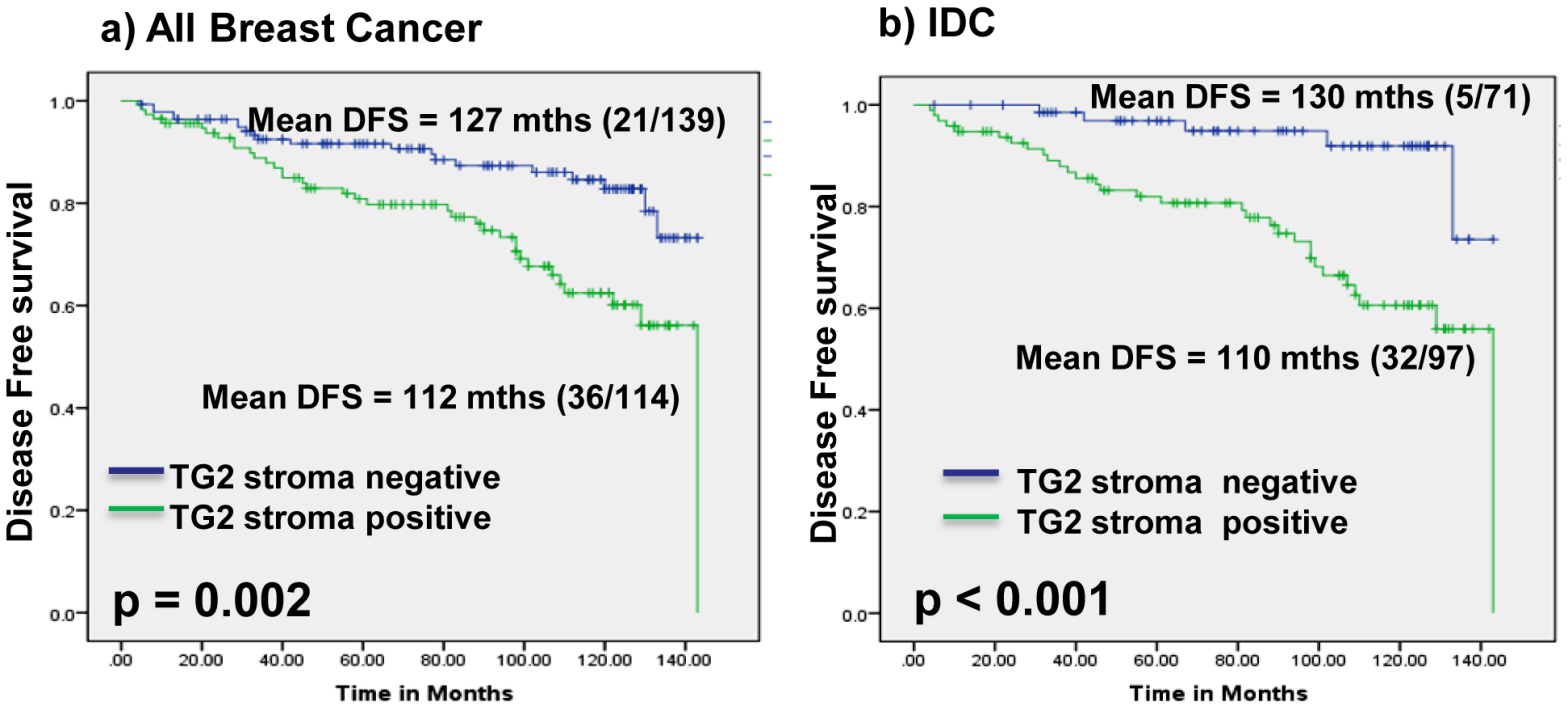
Kaplan Meier Survival Analysis. **Panel** (**a**) Kaplan Meier survival analysis in breast cancer patients. Panel shows breast cancer patients with TG2 overexpression in tumor stroma have significantly reduced DFS (mean DFS = 112 months, p = 0.002) as compared to patients with low or no detectable expression of stromal TG2 (mean DFS = 127 months). **Panel** (**b**) shows patients with IDCs of the breast demonstrating stromal TG2 overexpression have reduced DFS (mean DFS = 110 months, p<0.001) in comparison with patients showing lower TG2 expression in tumor stroma (mean DFS = 130 months).

Follow up data of 253 breast cancer patients for up to 12 years was used to assess the prognostic relevance of TG2 for predicting Disease free survival (DFS) including both loco-regional recurrence and distant metastasis. Recurrence (loco-regional/distant metastasis) was observed in 57 breast cancer patients including DCIS (n = 15), IDC (n = 37) and (ILC = 5) over a time period of 4–143 months. Kaplan-Meier survival analysis showed significantly reduced DFS of breast cancer patients showing TG2 accumulation in stroma (mean DFS = 112 months, p = 0.002) in comparison with patients showing lower expression (mean DFS = 127 months, Figure 4a, Table 3). Among the clinicopathological parameters, T-stage (p<0.001), nodal status (p = 0.001), and histology grade (p = 0.002) correlated with reduced DFS in breast cancers (Table 3). Among IDC patients, increased TG2 expression in tumor stroma correlated significantly with reduced DFS (mean DFS = 110 months) in comparison with patients showing lower stromal TG2 (mean DFS = 130 months, p<0.001, Figure 4b). All the 5 ILCs showing reduced DFS showed increased TG2 accumulation in tumor stroma. However, Kaplan Meier analysis could not be performed for ILC due to the small sample size. No significant association of cytoplasmic TG2 overexpression with recurrence was observed in all breast cancers analyzed as well as in DCIS and IDCs (Figure S1a–c).

**Table 3:** Survival analysis of Breast cancer patients.

	Kaplan Meier Survival analysis (p-value)	Multivariate Cox regression analysis (p-value)	Hazard’s Ratio (H.R.)	95% C.I.
**All breast cancer cases**				
Age	0.075	**–**	**–**	**–**
**T-stage**	**<0.001**	**0.001**	**5.1**	**(1.9**–**13.3)**
**Nodal Status**	**0.001**	**–**	**–**	**–**
**ER status**	**0.040**	**0.029**	**0.45**	**(0.3–0.9)**
PR status	0.173	**–**	**–**	**–**
**Grade**	**0.002**	**0.034**	**4.89**	**(1.1**–**21.2)**
TG2 (cytoplasm^+^)	0.157	**–**	**–**	**–**
**TG2 (Stroma^+^)**	**0.002**	**0.014**	**2.70**	**(1.2–5.9)**
**IDCs**				
Age	0.577	**–**	**–**	**–**
**T-stage**	**<0.001**	**<0.001**	**5.26**	**(2.1–13.7)**
**Nodal Status**	**0.006**	**–**	**–**	**–**
**Grade**	**0.002**	**–**	**–**	**–**
**ER status**	**0.010**	**0.008**	**0.361**	**(0.2**–**0.7)**
PR status	**0.076**	**–**	**–**	**–**
TG2 (cytoplasm^+^)	0.228	**–**	**–**	**–**
**TG2 (Stroma^+^)**	**<0.001**	**0.006**	**3.79**	**(1.4–9.8)**

In multivariate Cox regression analysis using TG2 overexpression (cytoplasm or stroma), age, ER/PR status, tumor stage, grade and nodal status as variables in the model, TG2 accumulation in tumor stroma (p = 0.014, Hazard’s ratio, H.R. = 2.7, 95% C.I. = 1.2–5.9), T-stage (p = 0.001) and grade (p = 0.034) emerged as independent factors associated with poor prognosis of breast cancer patients (Table 3). In IDC, TG2 stromal accumulation was associated with poor prognosis in a Cox multivariate analysis (p = 0.006, H.R. = 3.79, 95% C.I. = 1.4–9.8) and tumor stage (p<0.001, HR = 5.26, 95% C.I. = 2.1–13.7, Table 3).

### Evaluation of TG2 Transamidating Activity in Invasive Ductal Carcinomas (IDCs)

**Figure 5:**
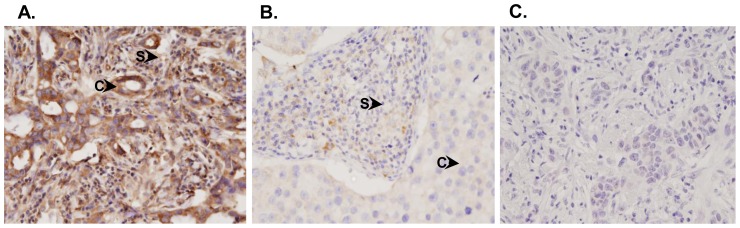
Immunohistochemistry of N-epsilon gamma-glutamyl lysine amino residues in IDC tissues. Panel shows (i) strong immunostaining for N-epsilon gamma-glutamyl lysine amino residues in both cytoplasm and stroma of IDC that showed strong TG2 staining; (ii) faint cytoplasmic but no staining for N-epsilon gamma-glutamyl lysine amino residues in stroma of IDCs that showed weak TG2 staining and (iii) negative control showing no TG2 immunostaining in either cytoplasm or stroma of IDCs (Original Magnification X400).

To evaluate the transamidating activity of TG2 overexpression in stroma/cytoplasm of IDCs, we determined the expression of N-epsilon gamma-glutamyl lysine amino residues in representative tissue sections of IDCs showing either low or high scores of stromal TG2 immunostaining. Of the 40 IDCs of the breast demonstrating high immunostaining scores of stromal TG2, 34 cases (85%) showed stromal staining of N-epsilon gamma-glutamyl lysine amino residues (Figure 5A). Interestingly, all IDCs (n = 35) showing low stromal/cytoplasmic TG2 staining also showed weak immunostaining for N-epsilon gamma-glutamyl lysine amino residues in cytoplasm and stroma of IDCs (Figure 5B). No immunostaining was observed either in cytoplasm or stroma of breast cancer tissues used as negative controls (Figure 5C). A correlation (R = 0.671, p = 0.016) was observed for co-localization of TG2 and N-epsilon gamma-glutamyl lysine amino residues immunostaining in stroma in tissue sections of IDCs.

### Evaluation of Phospho-FAK and Phospho-ERK1+ ERK2 in IDCs Showing Overexpression of Stromal TG2

To determine the effect of TG2 on activation of integrin dependent downstream cell signaling in IDCs showing overexpression of stromal TG2, we performed immunohistochemical analysis of phospho-FAK (Y397) and phospho-ERK1+ ERK2 (phospho T202+ T185+ Y187) in representative tissue sections. Our results of phospho-FAK (Y397) showed strong nuclear staining in all IDC sections analyzed, whereas no immunostaining in nucleus/cytoplasm was observed in IDC tissue sections used as negative controls (Figure S2A,i–iii). No significant difference in the nuclear expression for phospho-FAK (Y397) was observed among these IDC sections. Immunohistochemistry of phospho-ERK1+ERK2 (phospho T202+ T185+ Y187) showed no detectable immunostaining in IDCs showing overexpression of stromal TG2. However, thyroid cancer tissue sections used as a positive controls showed strong nuclear expression of phospho-ERK1+ERK2 (phospho T202+ T185+ Y187). Negative control tissue sections showed no immunostaining in cancer cells (Figure S2B, i–iii).

## Discussion

The development of metastasis poses a major clinical problem in treatment of breast cancer patients leading to poor cancer free survival. Identification of molecular markers which can predict recurrence is imperative for developing effective therapies for more effective disease management. Our study provides clinical evidence that TG2 expression is up-regulated in the primary tumor of patients likely to develop distant metastasis. The majority of IDC patients showed TG2 accumulation mainly in tumor stroma. Notably, significant association of stromal TG2 in IDC was observed with clinicopathologic features including increased tumor size, grade, stage and nodal metastasis which contribute to aggressive phenotype in these patients. Further, our results clearly demonstrated accumulation of TG2 in tumor stroma associated with loco-regional recurrence, distant metastasis, and hence decreased DFS of IDC patients. Our findings are supported by the report of Girgoriev *et al*., [32], which showed stromal TG2 expression in 50% of the human breast carcinomas, while 15% cases stained positive for cytoplasmic TG2; however the correlation of TG2 with disease outcome was not assessed in this study. Mehta *et al*., [33] reported TG2 expression was significantly higher in lymph nodes than in primary breast tumors, but their study was limited by a small size of 30 cases only and no follow up data were provided. Further in another independent study by same group, Mangala *et al*., [34] compared stromal TG2 expression in 189 early stage breast cancer cases and demonstrated significant association of higher stromal TG2 expression with negative lymph nodes (p<0.001). Although, authors reported median follow-up of 4 years for these patients with negative lymph nodes, statistical analysis including multivariate Cox regression for evaluating prognosis in breast cancer patients was not provided. Thus, our study assumes importance as the first report demonstrating association of TG2 accumulation in tumor stroma with poor disease outcome and its significant association with aggressive features such as increasing tumor size, grade, stage, lymph node and distal metastasis in a large cohort of breast cancers with special emphasis on invasive ductal carcinomas.

TG2 overexpression in metastatic breast cancer promotes apoptosis-resistance phenotype, cell migration and invasion by initiating integrin-mediated cell attachment and cell survival signalling pathways [14], [35]–[37]. A review of TG2 expression in other human cancers revealed overexpression of TG2 in pancreatic tumor cells associated with nodal metastasis, lymphovascular invasion and poor overall patient survival [38]. Further, TG2 overexpression in ovarian cancer patients was associated with poor overall survival [39], [40]. Increased expression of TG2 in ovarian cancer cells enhanced their adhesion to fibronectin and promoted directional cell migration, whereas knockdown of TG2 showed diminished tumor dissemination on the peritoneal surface and in mesentery in an intraperitoneal ovarian xenograft mouse model [41]–[44].

Extra-cellular TG2 along with β1 and β3 integrins serves as a co-receptor for fibronectin [14], [27], [45]. Interestingly, this integrin mediated interaction of TG2 and fibronectin promotes adhesion, migration, and spreading of cells on fibronectin-coated surfaces and is independent of the TG2 enzymatic activity [14], [27], [45]. TG2 in ECM associates with integrins inducing activation of anti-apoptotic protein Bcl-2, focal adhesion kinase (FAK) dependent signal transduction pathways including PI3K/Akt, and Ras/Erk, pathways which contribute to cancer aggressiveness [46]. Moreover, TG2 overexpression in ECM leads to increased accumulation of matrix bound transforming growth factor beta 1(TGF-β1), both in vitro and in vivo [27], [28], [47]. TG2 expression signals the onset of EMT in epithelial cells and contributes to their increased survival and metastatic potential [27], [28], [47]. The association of stromal TG2 with lymph nodal metastasis in breast cancer patients in our study provides clinical evidence in support of its utility as a marker of metastatic potential in these patients. The mechanistic basis of aberrant stromal TG2 expression contribution to EMT and metastatic capabilities of breast cancers warrants investigation in future studies.

Our results also demonstrated overexpression of N-epsilon gamma-glutamyl lysine amino residues in cytoplasm and stroma of IDCs demonstrating presence of active TG2 in these breast cancers. Notably, most of these breast cancer cases had poor prognosis indicating a plausible role of active TG2 in stroma in recurrence among breast cancer patients. However, lack of significant difference of phospho-FAK expression and absence of phospho-ERK in IDCs showing overexpression of stromal TG2 suggests that stromal TG2 may not activate integrins. Taken together our results suggest the crosslinking function of stromal TG2 might be important in IDCs of breast.

## Conclusions

Our study clearly demonstrates the clinical significance of stromal TG2 overexpression in breast IDCs and may serve as an independent risk factor for identifying patients with high risk of recurrence and metastasis. These patients can be followed more closely and managed appropriately by selecting other treatment modalities and thereby potentially reducing the morbidity due to recurrence. Further, it may also help avoid overtreatment of patients at low risk of disease recurrence reducing harmful side effects of therapy and reduce the economic burden on health care providers as well.

## Supporting Information

Figure S1 **Kaplan Meier Survival Analysis.** Panel shows Kaplan Meier survival analysis in (a) all breast cancer patients; (b) DCIS; (c) IDC depicting no significant difference in mean DFS of patients showing cytoplasmic TG2 staining in all the three panels.
(TIF)

Figure S2 **(A) Immunohistochemistry of phospho-FAK (phospho Y397)** **in IDC tissues**. Panel shows strong nuclear immunostaining of phospho-FAK (phospho Y397) in (i) IDC tissue section that showed strong TG2 immunostaining in stroma; (ii) IDC tissue section that showed weak TG2 immunostaining in stroma and (iii) negative control showing no immunostaining in nucleus/cytoplasm of tissue section (Original Magnification X400). **(B) Immunohistochemistry of in anti-ERK1+ERK2 (phospho T202+ T185+ Y187). IDC tissues**. Panel shows (i) IDC tissue section showing no immunostaining anti-ERK1+ERK2 (phospho T202+ T185+ Y187) in nucleus/cytoplasm of breast cancer cells; (ii) thyroid cancer tissue section used as positive control showed strong nuclear staining phospho-ERK and (iii) thyroid cancer tissue section used as negative control showing no immunostaining in nucleus/cytoplasm of thyroid cancer cells (Original Magnification X400).
(TIF)

## References

[1] Siegel R, Naishadham D, Jemal A (2012) Cancer statistics, 2012. CA Cancer J Clin 62: 10–29. 22237781 10.3322/caac.20138

[2] Brewster AM, Hortobagyi GN, Broglio KR, Kau SW, Santa-Maria CA, et al (2008) Residual risk of breast cancer recurrence 5 years after adjuvant therapy. J Natl Cancer Inst 100: 1179–1183. 18695137 10.1093/jnci/djn233 PMC6592411

[3] Weigelt B, Geyer FC, Reis-Filho JS (2010) Histological types of breast cancer: how special are they? Mol Oncol 4: 192–208. 20452298 10.1016/j.molonc.2010.04.004 PMC5527938

[4] Bertos NR, Park M (2011) Breast cancer - one term, many entities? J Clin Invest 121: 3789–3796. 21965335 10.1172/JCI57100 PMC3195465

[5] Almendro V, Fuster G (2011) Heterogeneity of breast cancer: etiology and clinical relevance. Clin Transl Oncol 13: 767–773. 22082639 10.1007/s12094-011-0731-9

[6] Fordyce CA, Patten KT, Fessenden TB, Defilippis R, Hwang ES, et al (2012) Cell-extrinsic consequences of epithelial stress: activation of protumorigenic tissue phenotypes. Breast Cancer Res 14: R155. 23216814 10.1186/bcr3368 PMC3786321

[7] Kalluri R, Zeisberg M (2006) Fibroblasts in cancer. Nat Rev Cancer 6: 392–401. 16572188 10.1038/nrc1877

[8] Olumi AF, Grossfeld GD, Hayward SW, Carroll PR, Tlsty TD, et al (1999) Carcinoma-associated fibroblasts direct tumor progression of initiated human prostatic epithelium. Cancer Res 59: 5002–5011. 10519415 10.1186/bcr138 PMC3300837

[9] Orimo A, Gupta PB, Sgroi DC, Arenzana-Seisdedos F, Delaunay T, et al (2005) Stromal fibroblasts present in invasive human breast carcinomas promote tumor growth and angiogenesis through elevated SDF-1/CXCL12 secretion. Cell 121: 335–348. 15882617 10.1016/j.cell.2005.02.034

[10] Tlsty TD, Coussens LM (2006) Tumor stroma and regulation of cancer development. Annu Rev Pathol 1: 119–150. 18039110 10.1146/annurev.pathol.1.110304.100224

[11] Lisanti MP, Martinez-Outschoorn UE, Chiavarina B, Pavlides S, Whitaker-Menezes D, et al (2010) Understanding the “lethal” drivers of tumor-stroma co-evolution: emerging role(s) for hypoxia, oxidative stress and autophagy/mitophagy in the tumor micro-environment. Cancer Biol Ther 10: 537–542. 20861671 10.4161/cbt.10.6.13370 PMC3040943

[12] Fiaschi T, Chiarugi P (2012) Oxidative stress, tumor microenvironment, and metabolic reprogramming: a diabolic liaison. Int J Cell Biol 2012: 762825. 22666258 10.1155/2012/762825 PMC3361160

[13] Kotsakis P, Griffin M (2007) Tissue transglutaminase in tumour progression: friend or foe? Amino Acids 33: 373–384. 17581697 10.1007/s00726-007-0516-1

[14] Mehta K, Kumar A, Kim HI (2010) Transglutaminase 2: a multi-tasking protein in the complex circuitry of inflammation and cancer. Biochem Pharmacol 80: 1921–1929. 20599779 10.1016/j.bcp.2010.06.029

[15] Chhabra A, Verma A, Mehta K (2009) Tissue transglutaminase promotes or suppresses tumors depending on cell context. Anticancer Res 29: 1909–1919. 19528447

[16] Mangala LS, Mehta K (2005) Tissue transglutaminase (TG2) in cancer biology. Prog Exp Tumor Res 38: 125–138. 15746533 10.1159/000084237

[17] Kumar A, Xu J, Brady S, Gao H, Yu D, et al (2010) Tissue transglutaminase promotes drug resistance and invasion by inducing mesenchymal transition in mammary epithelial cells. PLoS One 5: e13390. 20967228 10.1371/journal.pone.0013390 PMC2953521

[18] Kumar A, Gao H, Xu J, Reuben J, Yu D, et al (2011) Evidence that aberrant expression of tissue transglutaminase promotes stem cell characteristics in mammary epithelial cells. PLoS One 6: e20701. 21687668 10.1371/journal.pone.0020701 PMC3110765

[19] Kumar A, Xu J, Sung B, Kumar S, Yu D, et al (2012) Evidence that GTP-binding domain but not catalytic domain of transglutaminase 2 is essential for epithelial-to-mesenchymal transition in mammary epithelial cells. Breast Cancer Res 14: R4. 22225906 10.1186/bcr3085 PMC3496119

[20] Cao L, Shao M, Schilder J, Guise T, Mohammad KS, et al (2012) Tissue transglutaminase links TGF-beta, epithelial to mesenchymal transition and a stem cell phenotype in ovarian cancer. Oncogene 31: 2521–2534. 21963846 10.1038/onc.2011.429

[21] Lin CY, Tsai PH, Kandaswami CC, Chang GD, Cheng CH, et al (2011) Role of tissue transglutaminase 2 in the acquisition of a mesenchymal-like phenotype in highly invasive A431 tumor cells. Mol Cancer 10: 87. 21777419 10.1186/1476-4598-10-87 PMC3150327

[22] Park KS, Kim DS, Ko C, Lee SJ, Oh SH, et al (2011) TNF-alpha mediated NF-kappaB activation is constantly extended by transglutaminase 2. Front Biosci (Elite Ed) 3: 341–354. 21196314 10.2741/e249

[23] Mann AP, Verma A, Sethi G, Manavathi B, Wang H, et al (2006) Overexpression of tissue transglutaminase leads to constitutive activation of nuclear factor-kappaB in cancer cells: delineation of a novel pathway. Cancer Res 66: 8788–8795. 16951195 10.1158/0008-5472.CAN-06-1457

[24] Kuo TF, Tatsukawa H, Kojima S (2011) New insights into the functions and localization of nuclear transglutaminase 2. FEBS J 278: 4756–4767. 22051117 10.1111/j.1742-4658.2011.08409.x

[25] Mishra S, Saleh A, Espino PS, Davie JR, Murphy LJ (2006) Phosphorylation of histones by tissue transglutaminase. J Biol Chem 281: 5532–5538. 16407273 10.1074/jbc.M506864200

[26] Jeon JH, Choi KH, Cho SY, Kim CW, Shin DM, et al (2003) Transglutaminase 2 inhibits Rb binding of human papillomavirus E7 by incorporating polyamine. EMBO J 22: 5273–5282. 14517264 10.1093/emboj/cdg495 PMC204478

[27] Belkin AM (2011) Extracellular TG2: emerging functions and regulation. FEBS J 278: 4704–4716. 21902810 10.1111/j.1742-4658.2011.08346.x PMC3228878

[28] Wang Z, Griffin M (2012) TG2, a novel extracellular protein with multiple functions. Amino Acids 42: 939–949. 21818567 10.1007/s00726-011-1008-x

[29] Kang SK, Yi KS, Kwon NS, Park KH, Kim UH, et al (2004) Alpha1B-adrenoceptor signaling and cell motility: GTPase function of Gh/transglutaminase 2 inhibits cell migration through interaction with cytoplasmic tail of integrin alpha subunits. J Biol Chem 279: 36593–36600. 15220331 10.1074/jbc.M402084200

[30] Baek KJ, Kang S, Damron D, Im M (2001) Phospholipase Cdelta1 is a guanine nucleotide exchanging factor for transglutaminase II (Galpha h) and promotes alpha 1B-adrenoreceptor-mediated GTP binding and intracellular calcium release. J Biol Chem 276: 5591–5597. 11087745 10.1074/jbc.M008252200

[31] Tripathi SC, Matta A, Kaur J, Grigull J, Chauhan SS, et al (2011) Overexpression of prothymosin alpha predicts poor disease outcome in head and neck cancer. PLoS One 6: e19213. 21573209 10.1371/journal.pone.0019213 PMC3088661

[32] Grigoriev MY, Suspitsin EN, Togo AV, Pozharisski KM, Ivanova OA, et al (2001) Tissue transglutaminase expression in breast carcinomas. J Exp Clin Cancer Res 20: 265–268. 11484985

[33] Mehta K, Fok J, Miller FR, Koul D, Sahin AA (2004) Prognostic significance of tissue transglutaminase in drug resistant and metastatic breast cancer. Clin Cancer Res 10: 8068–8076. 15585642 10.1158/1078-0432.CCR-04-1107

[34] Mangala LS, Arun B, Sahin AA, Mehta K (2005) Tissue transglutaminase-induced alterations in extracellular matrix inhibit tumor invasion. Mol Cancer 4: 33. 16153302 10.1186/1476-4598-4-33 PMC1224867

[35] Mangala LS, Fok JY, Zorrilla-Calancha IR, Verma A, Mehta K (2007) Tissue transglutaminase expression promotes cell attachment, invasion and survival in breast cancer cells. Oncogene 26: 2459–2470. 17043648 10.1038/sj.onc.1210035

[36] Kim DS, Park SS, Nam BH, Kim IH, Kim SY (2006) Reversal of drug resistance in breast cancer cells by transglutaminase 2 inhibition and nuclear factor-kappaB inactivation. Cancer Res 66: 10936–10943. 17108131 10.1158/0008-5472.CAN-06-1521

[37] Oh K, Ko E, Kim HS, Park AK, Moon HG, et al (2011) Transglutaminase 2 facilitates the distant hematogenous metastasis of breast cancer by modulating interleukin-6 in cancer cells. Breast Cancer Res 13: R96. 21967801 10.1186/bcr3034 PMC3262209

[38] Verma A, Wang H, Manavathi B, Fok JY, Mann AP, et al (2006) Increased expression of tissue transglutaminase in pancreatic ductal adenocarcinoma and its implications in drug resistance and metastasis. Cancer Res 66: 10525–10533. 17079475 10.1158/0008-5472.CAN-06-2387

[39] Singer CF, Hudelist G, Walter I, Rueckliniger E, Czerwenka K, et al (2006) Tissue array-based expression of transglutaminase-2 in human breast and ovarian cancer. Clin Exp Metastasis 23: 33–39. 16826431 10.1007/s10585-006-9015-0

[40] Hwang JY, Mangala LS, Fok JY, Lin YG, Merritt WM, et al (2008) Clinical and biological significance of tissue transglutaminase in ovarian carcinoma. Cancer Res 68: 5849–5858. 18632639 10.1158/0008-5472.CAN-07-6130 PMC2547344

[41] Khanna M, Chelladurai B, Gavini A, Li L, Shao M, et al (2011) Targeting ovarian tumor cell adhesion mediated by tissue transglutaminase. Mol Cancer Ther 10: 626–636. 21330459 10.1158/1535-7163.MCT-10-0912

[42] Cao L, Petrusca DN, Satpathy M, Nakshatri H, Petrache I, et al (2008) Tissue transglutaminase protects epithelial ovarian cancer cells from cisplatin-induced apoptosis by promoting cell survival signaling. Carcinogenesis 29: 1893–1900. 18667446 10.1093/carcin/bgn158 PMC2556973

[43] Satpathy M, Cao L, Pincheira R, Emerson R, Bigsby R, et al (2007) Enhanced peritoneal ovarian tumor dissemination by tissue transglutaminase. Cancer Res 67: 7194–7202. 17671187 10.1158/0008-5472.CAN-07-0307

[44] Satpathy M, Shao M, Emerson R, Donner DB, Matei D (2009) Tissue transglutaminase regulates matrix metalloproteinase-2 in ovarian cancer by modulating cAMP-response element-binding protein activity. J Biol Chem 284: 15390–15399. 19324884 10.1074/jbc.M808331200 PMC2708835

[45] Akimov SS, Krylov D, Fleischman LF, Belkin AM (2000) Tissue transglutaminase is an integrin-binding adhesion coreceptor for fibronectin. J Cell Biol 148: 825–838. 10684262 10.1083/jcb.148.4.825 PMC2169362

[46] Verma A, Guha S, Wang H, Fok JY, Koul D, et al (2008) Tissue transglutaminase regulates focal adhesion kinase/AKT activation by modulating PTEN expression in pancreatic cancer cells. Clin Cancer Res 14: 1997–2005. 18381937 10.1158/1078-0432.CCR-07-1533

[47] Collighan RJ, Griffin M (2009) Transglutaminase 2 cross-linking of matrix proteins: biological significance and medical applications. Amino Acids 36: 659–670. 18982407 10.1007/s00726-008-0190-y

