# Supplementary figures and images for: Transglutaminase 2 Overexpression in Tumor Stroma Identifies Invasive Ductal Carcinomas of Breast at High Risk of Recurrence
Source: PLoS One. 2013 Sep 13;8(9):e74437. doi: 10.1371/journal.pone.0074437 (PMC3772876; doi:10.1371/journal.pone.0074437)

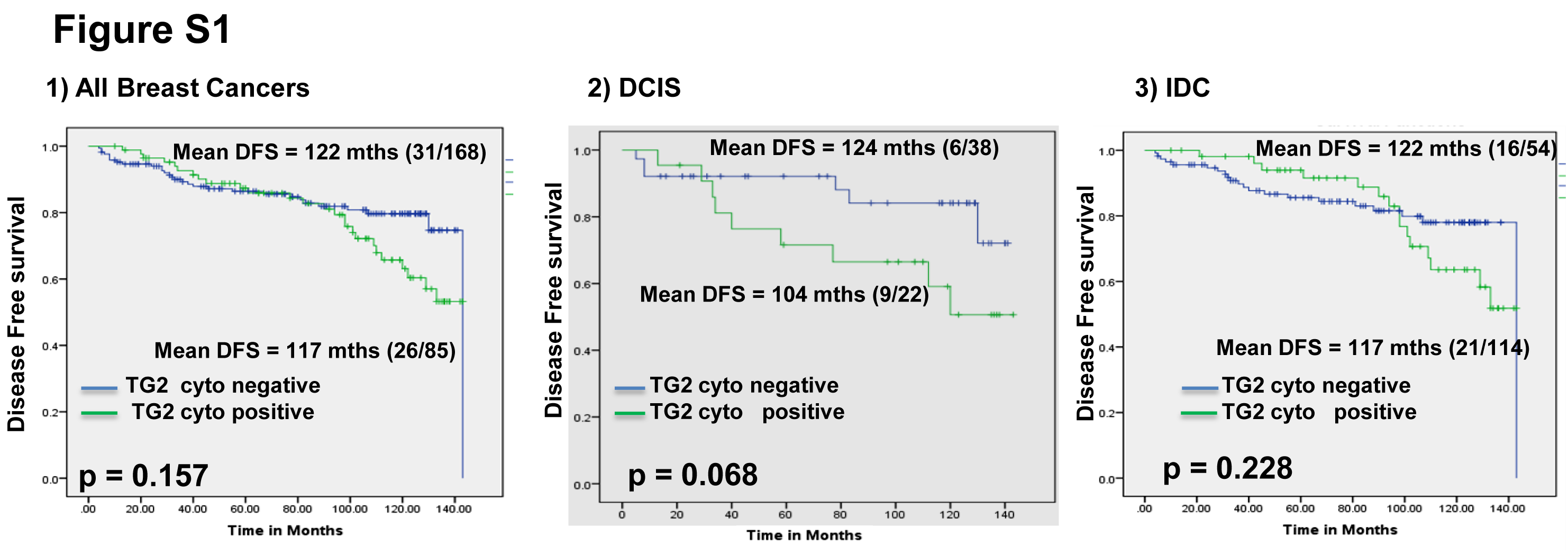

Supplement: Figure S1 — Kaplan Meier Survival Analysis. Panel shows Kaplan Meier survival analysis in (a) all breast cancer patients; (b) DCIS; (c) IDC depicting no significant difference in mean DFS of patients showing cytoplasmic TG2 staining in all the three panels. (TIF) [file pone.0074437.s001.tif]

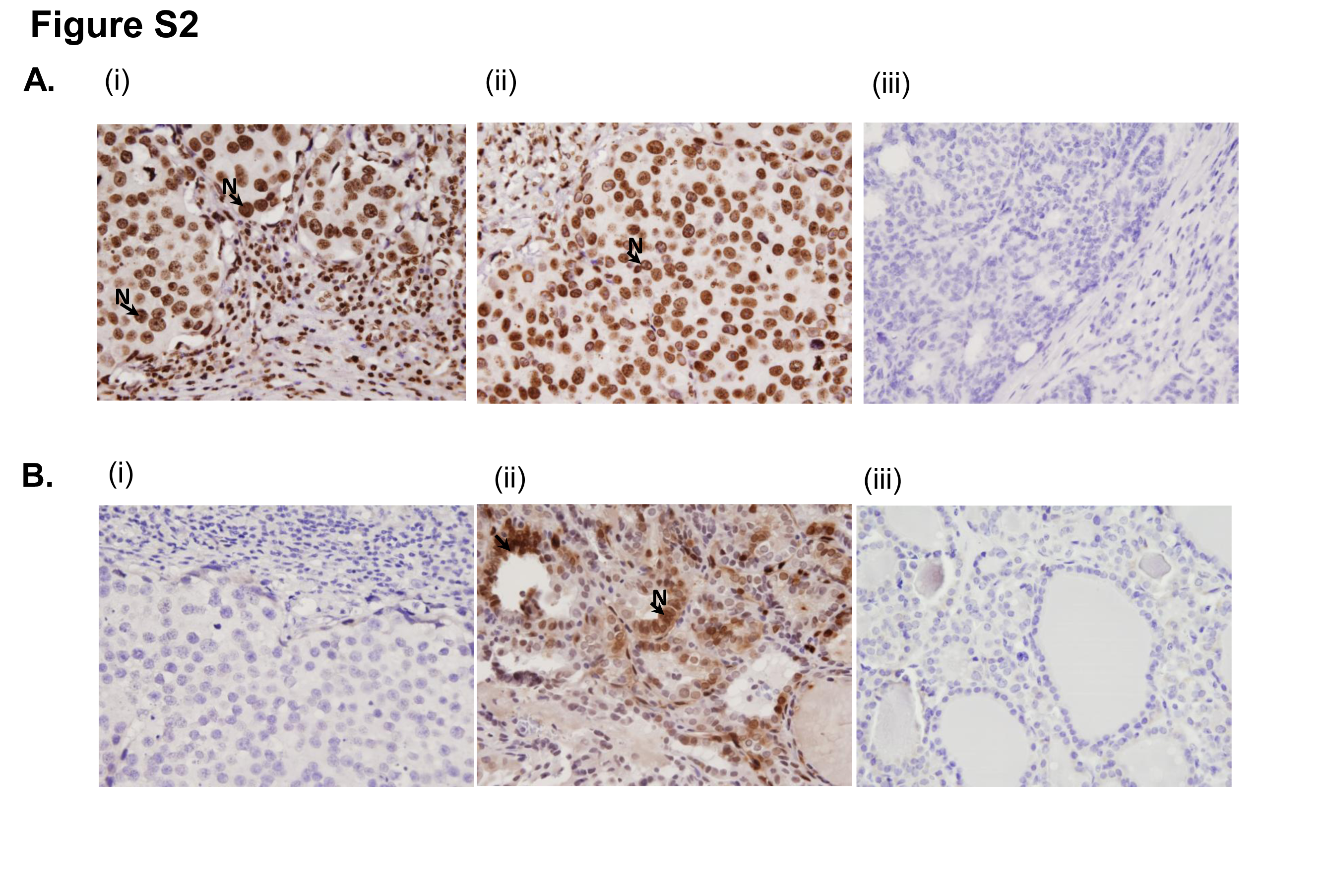

Supplement: Figure S2 — (A) Immunohistochemistry of phospho-FAK (phospho Y397) in IDC tissues. Panel shows strong nuclear immunostaining of phospho-FAK (phospho Y397) in (i) IDC tissue section that showed strong TG2 immunostaining in stroma; (ii) IDC tissue section that showed weak TG2 immunostaining in stroma and (iii) negative control showing no immunostaining in nucleus/cytoplasm of tissue section (Original Magnification X400). (B) Immunohistochemistry of in anti-ERK1+ERK2 (phospho T202+ T185+ Y187). IDC tissues. Panel shows (i) IDC tissue section showing no immunostaining anti-ERK1+ERK2 (phospho T202+ T185+ Y187) in nucleus/cytoplasm of breast cancer cells; (ii) thyroid cancer tissue section used as positive control showed strong nuclear staining phospho-ERK and (iii) thyroid cancer tissue section used as negative control showing no immunostaining in nucleus/cytoplasm of thyroid cancer cells (Original Magnification X400). (TIF) [file pone.0074437.s002.tif]
